# Insights into the structure of *Escherichia coli* outer membrane as the target for engineering microbial cell factories

**DOI:** 10.1186/s12934-021-01565-8

**Published:** 2021-03-20

**Authors:** Jianli Wang, Wenjian Ma, Xiaoyuan Wang

**Affiliations:** 1 State Key Laboratory of Food Science and Technology, Jiangnan University, 1800 Lihu Avenue, Wuxi, 214122 China; 2 International Joint Laboratory On Food Safety, Jiangnan University, Wuxi, 214122 China; 3 Science Center for Future Foods, Jiangnan University, Wuxi, 214122 China; 4 Key Laboratory of Industrial Biotechnology, Ministry of Education, School of Biotechnology, Jiangnan University, Wuxi, 214122 China

**Keywords:** Outer membrane, Lipopolysaccharide, Exopolysaccharide, Flagella, Fimbria, Membrane engineering, Poly-3-hydroxybutyrate, Inclusion bodies, Microbial cell factories, *Escherichia coli*

## Abstract

*Escherichia coli* is generally used as model bacteria to define microbial cell factories for many products and to investigate regulation mechanisms. *E. coli* exhibits phospholipids, lipopolysaccharides, colanic acid, flagella and type I fimbriae on the outer membrane which is a self-protective barrier and closely related to cellular morphology, growth, phenotypes and stress adaptation. However, these outer membrane associated molecules could also lead to potential contamination and insecurity for fermentation products and consume lots of nutrients and energy sources. Therefore, understanding critical insights of these membrane associated molecules is necessary for building better microbial producers. Here the biosynthesis, function, influences, and current membrane engineering applications of these outer membrane associated molecules were reviewed from the perspective of synthetic biology, and the potential and effective engineering strategies on the outer membrane to improve fermentation features for microbial cell factories were suggested.

## Background

*Escherichia coli* is generally used as a model bacteria to define microbial cell factories for many products and to investigate regulation mechanisms. The engineering on metabolic pathway and regulatory factors always attracted our attention, and many effective strategies have indeed been achieved. Recently, the researches on membrane engineering to improve the efficiency of bacterial cell factories suggest the importance of outer membrane (OM). As we know, the uptake of nutrients and export of products both need transmembrane transport, and the OM defined as the effective permeability barrier owns complex nanomachines spanning the cell envelope. The OM is also responsible for maintaining cell morphology and cell sizes [1–3]. In addition, regulation of cellular metabolism may change in response to changes in the structure of the OM. Therefore, better understanding the OM molecules are very important for engineering or optimizing the microbial producers.

**Fig. 1:**
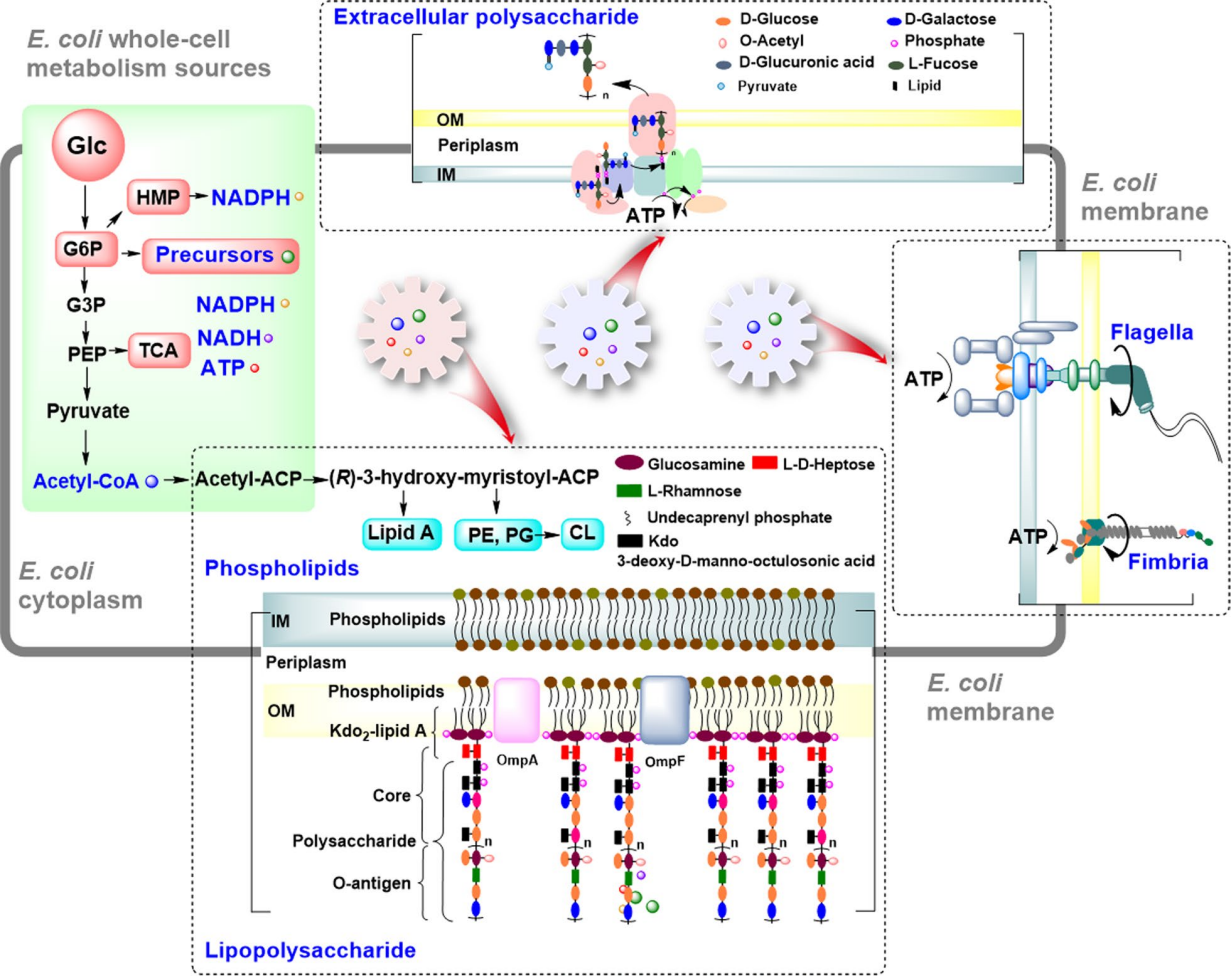
Different molecules of the OM in *E. coli*. The cell envelope of *E. coli* has two distinct membranes: the OM and the IM. Structurally, OM is composed of phospholipids in the periplasmic leaflet and LPS in the external leaflet. LPS contain Kdo_2_-lipid A, core and O-antigen, and the latter two form the polysaccharide portion of LPS. The biosynthesis of sugar groups in polysaccharide of LPS are derived from glucose 6-phosphate, an intermediate of glycolysis. The polymerized CA is secreted into the surface of the OM in *E. coli* K-12. CA contains six sugar groups also derived from the intermediates of glucose metabolic pathways. The structural model of flagella and type I fimbriae exhibited on the membrane in *E. coli* K-12. The structure of flagella and type I fimbria are transmembrane anchored in the membrane with the flagellar filament and mycelium outside. The biosynthesis or function of LPS, CA, flagella and fimbria all need co-factors ATP

The OM of *E. coli* plays important roles not only on the cell morphology, division, phenotypes, and stress adaptations, but also on the intracellular metabolism. In *E. coli*, there are two distinct membranes: the OM and the inner membrane (IM) (Fig. 1) [4, 5]. And the envelope defines cell shape and allows the cell to sustain large mechanical loads such as turgor pressure [6]. It is widely believed that the OM could prevent the entry of hydrophobic compounds and large hydrophilic molecules, and is responsible for the intrinsic resistance of *E. coli* to antibiotics, detergents and dyes [7]. Recent report also demonstrated that the stiffness of *E. coli* cell envelope is largely due to the OM [6]. The covalently cross-linked cell wall underpins the mechanical properties of the envelope.

**Fig. 2:**
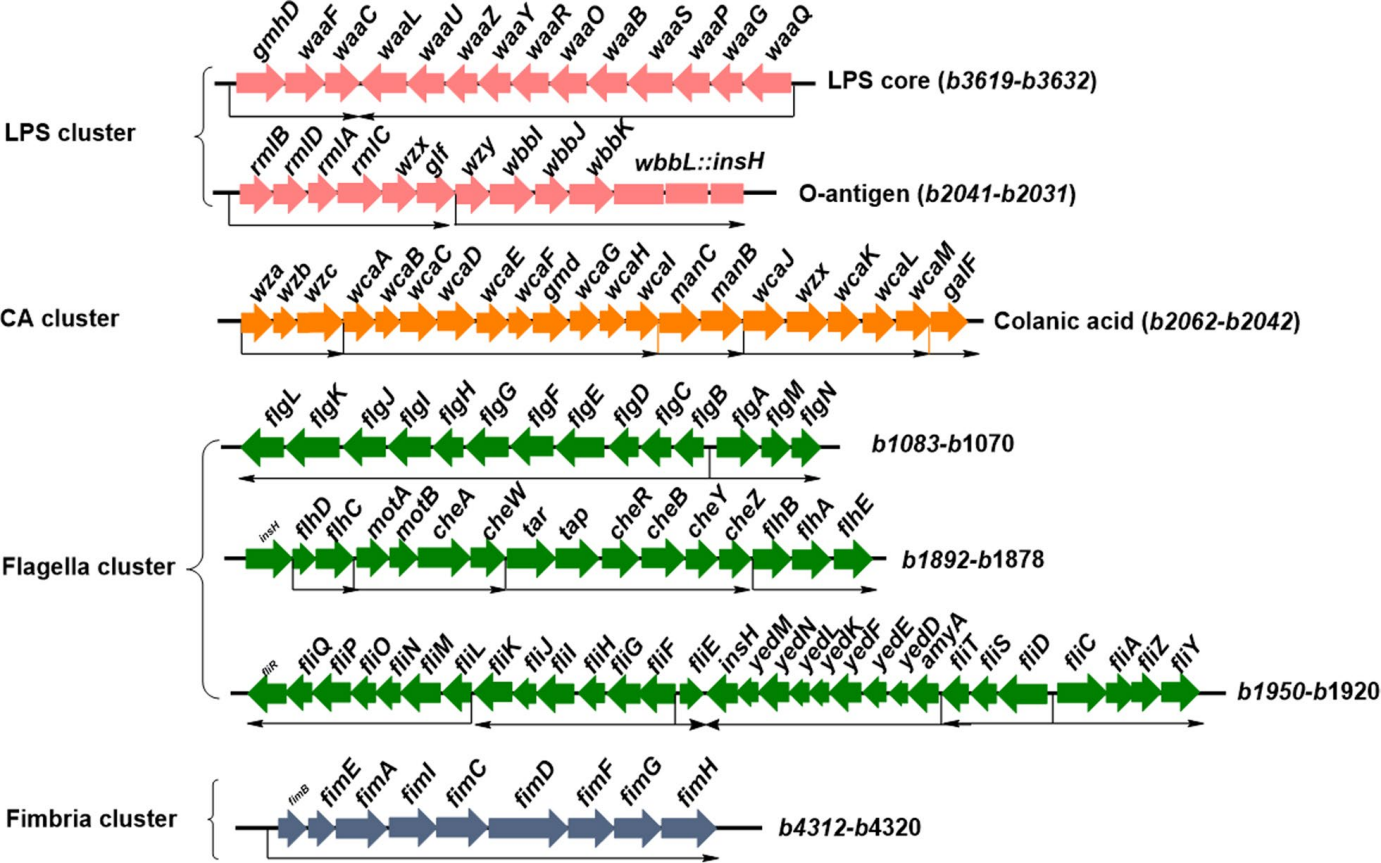
The involved gene clusters for LPS including core and O-antigen, CA, flagella and type I fimbria in *E. coli*

Structurally, *E. coli* exhibits OM proteins (Omps), phospholipids, lipopolysaccharide (LPS), exopolysaccharide (EPS), flagella and type I fimbriae on the OM with phospholipids in the periplasmic leaflet and LPS in the external leaflet, and various Omps populating this membrane (Fig. 1). The OmpC and OmpF are the two most important OM porin proteins in *E. coli*, and control the passage of small molecule solutes into the cell interior [8]. Another important porin OmpA plays a structural role in the integrity of the bacterial cell surface [9]. The polysaccharide portion of LPS, EPS, flagella and fimbria are non-essential structures, and the relevant genes are listed in different operons in *E. coli* (Fig. 2). LPS contributes to the stiffness of the OM and the structural integrity of bacteria [6]. There are ∼10^6^ LPS molecules and ∼10^7^ glycerophospholipids per *E. coli* cell [7]. Phospholipids consist of a glycerol molecule, a phosphate group, and two fatty acid moieties (except for cardiolipins) [10]. The phospholipids had been determined to be closely associated to cell division and DNA replication [11]. LPS is a negatively charged amphipathic molecule composed of three covalently linked moieties: lipid A, a proximally located hydrophobic anchor that serves as an endotoxin; a core oligosaccharide, and a long polysaccharide called O-antigen (Fig. 1) [5]. LPS is an endotoxin that could be recognized by immune cells as a pathogen-associated molecule and elicit a strong immune response [7, 12]. *E. coli* can also produce and export polysaccharides to cell envelope (Fig. 1) [8]. In addition to the O-antigen, secreted polysaccharides with long-chain (∼10^5^–10^6^ Da) in the form of polymers (EPSs), or cell-associated capsular polysaccharides (CPSs) could also envelop the cell in a hydrophilic layer known as the capsule [13]. The polysaccharides contribute to the self-protection for cells when response to the extreme stress environment [14, 15], but are generally non-essential under normal conditions. The biosynthesis of these long-chained polysaccharides for LPS and secreted polysaccharides consume a lot of carbon and energy sources.

In addition to the Omps, lipids and polysaccharides, *E. coli* cells also exhibit many flagella and type I fimbriae assembly on the envelope surface (Fig. 1), and they provide swimming and swarming motilities of cells [16]. Flagella and the Type 1 fimbriae of *E. coli* are filamentous surface organelles, which mediate bacterial adherence to biotic and abiotic surfaces, leading to formation of biofilm and colonization on infected hosts [17]. Motility is an important quality of many bacteria for exploring the environment for nutrients, escaping from predator grazing and moving away from detrimental physicochemical conditions. However, the production and the rotation of flagella and type I fimbria are both energy-demanding processes for the cell [18]. Flagellar synthesis imposes a cost of approximately 2% of the biosynthetic energy expenditure of the cell in *E. coli* [19].

All these OM components are critical to ensure the normal physical functions of OM. However, these native OM structures in *E. coli* could lead to lots of unpleasant features on industrial application such as cytotoxicity [12], immune response [7], adhesion [17], biofilm formation [20], antibiotic resistance [21] and host invasion [16, 21]. These might lead to unsafety and toxicity for many chemicals production, especially for edible or medical products such as amino acids, organic acids, inclusion bodies and others. The lipids including phospholipids and Kdo_2_-lipid A are related to membrane fluidity, stiffness, biorenewables tolerance and pathogenicity. Moreover, these non-essential membrane molecules biosynthesis may become cell burdens consuming lots of nutrients and energy sources, including co-factors NADPH, NADH, ATP, and intermediate metabolites derived from glucose metabolisms (Fig. 1). Notably, several studies have showed that metabolic engineering on the pathways of membrane biosynthesis could significantly improve polyhydroxyalkanoates (PHA) production (Table 1). Hence, systematic and comprehensive understanding of the OM biosynthesis and function is important for better metabolic engineering and application development in *E. coli*. Here, we reviewed the biosynthesis pathway, functions and engineering application of these OM components, and make a based meaningful foreshadowing for metabolic engineering to yield genetically defined better overproducers.

**Table 1:** Improving the features and efficiency of microbial cell factories by membrane engineering

Strains	Control strains	Strategies	Changes	Refs.
+ pssA	*E. coli* MG1655	Expressing *pssA*	Increasing tolerance and production of octanoic acid; the membrane thickness; growth rate	[45]
Mutants overexpressing *cti*	*E. coli* MG1655	Expressing *cti* from *Pseudomonas aeruginosa*	Increasing tolerance and production of octanoic acid	[46]
PALK (pMS3- pelB-cti)	*Mannheimia succiniciproducens*	Δ*ldhA*, Δ*pta*, Δ*ackA*, + *pelB*, + *cti* (*Pseudomonas aeruginosa*)	Reducing membrane fluidity; increasing tolerance and production of succinic acid	[47]
CAR015-37Almgs(pPlsb-plsc)	CAR015(pACYC184-M)	overexpressing *plsB* and *plsC*	2.9-fold increase of β-carotene (m 6.7 to 19.6 mg/g DCW)	[48]
CAR025-37Almgs(pPlsb-plsc)	CAR025(pACYC184-M)	overexpressing *plsB* and *plsC*	39% increase of β-carotene (from 31.8 to 44.2 mg/g DCW)	[48]

Membrane engineering to improve PHA content %wt (Mutant/control)				
*E. coli*				
WJW00 (pDXW-8-*phaCAB*)	W3110 (pDXW-8-*phaCAB*)	Truncating LPS by deleting *gmhD*	67.8%/22.4%	[2]
WJD00 (pDXW-8-*phaCAB*)	DH5α (pDXW-8-*phaCAB*)	Truncating LPS by deleting *gmhD*	78.6%/42%	[2]
WJJ00 (pDXW-8-*phaCAB*)	JM109 (pDXW-8-*phaCAB*)	Truncating LPS by deleting *gmhD*	84.8%/48.3%	[2]
JM109-*murC*2 (pBHR68)	JM109 (pBHR68)	Targeting on *murC* for peptidoglycan synthesis via sgRNAs of CRISPRi	84.3%/78.85%	[3]
JM109-*mraY4* (pBHR68)	JM109 (pBHR68)	Interfering *mraY* for peptidoglycan synthesis	88.4%/78.85%	[3]
JM109-*ftsW2* (pBHR68)	JM109 (pBHR68)	Interfering *ftsW* for peptidoglycan synthesis	90.88%/78.85%	[3]
JM109-*murE1* + *murD2*) (pBHR68)	JM109 (pBHR68)	Interfering *murE* and *murD* for peptidoglycan synthesis	90.29%/78.85%	[3]
JM109-(*ftsW1* + *ftsW4*) (pBHR68)	JM109 (pBHR68)	Interfering *ftsW* for peptidoglycan synthesis	92.66%/78.85%	[3]
*R. eutropha*				
HF39 DO10	H16	Interfering LPS biosynthesis by *Tn5* insertion in H16_A0803 (*hldA*)	38%/25%	[59]
*P. putida*				
WJPP03	KT2442	Deleting 76 genes relevant to flagella and pili	63.1%/45.7%	[116]

### Outer membrane proteins play important roles in stress resistance and cell wall rigidity

In *E. coli*, bout 50% of the OM mass consists of proteins are anchored to the membrane [9]. The major porins OmpF and OmpC are closely related to the OM permeability, and allow ions, nutrient molecules, amino acids and sugars across OM [8]. Although it has been proposed that OmpC and OmpF are required under some harsh conditions in Gram-negative bacteria, *E. coli* cells are able to grow in the absence of these porin proteins [22]. The total amount of OmpF and OmpC pores remains constant in the *E. coli* membrane and vary slightly in response to changes in environment [8]. Notably, higher osmolarity or acidic pH environment could lead to the decrease of OmpC and OmpF in order to balance the physiological homeostasis of bacteria under extreme osmolarity and pH conditions [23]. OmpC and OmpF are required for hyperosmotic adaptation at pHs above 8.0, but not below 8.0 [22]. The transport of arginine, lysine, and their decarboxylated products through OmpC and/or OmpF is essential for the survival of *E. coli* cells under extremely acidic conditions [24]. The pore size of OmpF is larger than that of OmpC, thereby allowing more solutes including noxious agents to diffuse into the cell through the OmpF channel [8]. There are at least 100,000 copies of OmpF per cell to form passive channels for translocation of hydrophilic solutes of < 600 Da across the OM [23].

OmpA protein with about 100, 000 copies per cell mainly functions in the integrity of the bacterial cell surface to maintain cell shape [9]. The OmpA plays important roles in anchoring of the OM to the bacterial cell wall with special interaction with peptidoglycan [25, 26], and maintain the position of the cell wall [27]. Because peptidoglycan is the major constituent of the bacterial cell wall and provides structural strength and controls cell shape, and its integrity is critical for bacterial survival [28]. Hence, the interaction of OmpA with the cell wall is believed to provide stability to the supramolecular assembly and result in cellular integrity [25]. In addition, OmpA also functions as an adhesin and invasin, participates in biofilm formation, acts as both an immune target and evasin, and serves as a receptor for several bacteriophages [29]. The *ompA*-deletion mutant was significantly more sensitive than that of its parent strain to sodium dodecyl sulfate (SDS), cholate, acidic environment, high osmolarity, and pooled human serum [30]. Therefore, Omps influence cell permeability, drug resistance, stress resistance and cell morphology. Engineering OmpC and OmpF may influence respond manners to acid stress; and engineering OmpA may influence cell morphology or cell wall rigidity. However, there is little reports on engineering Omps for building microbial cell factories.

### Kdo_2_-lipid A and phospholipids influence the pathogenicity and OM fluidity of ***E. coli***

**Fig. 3:**
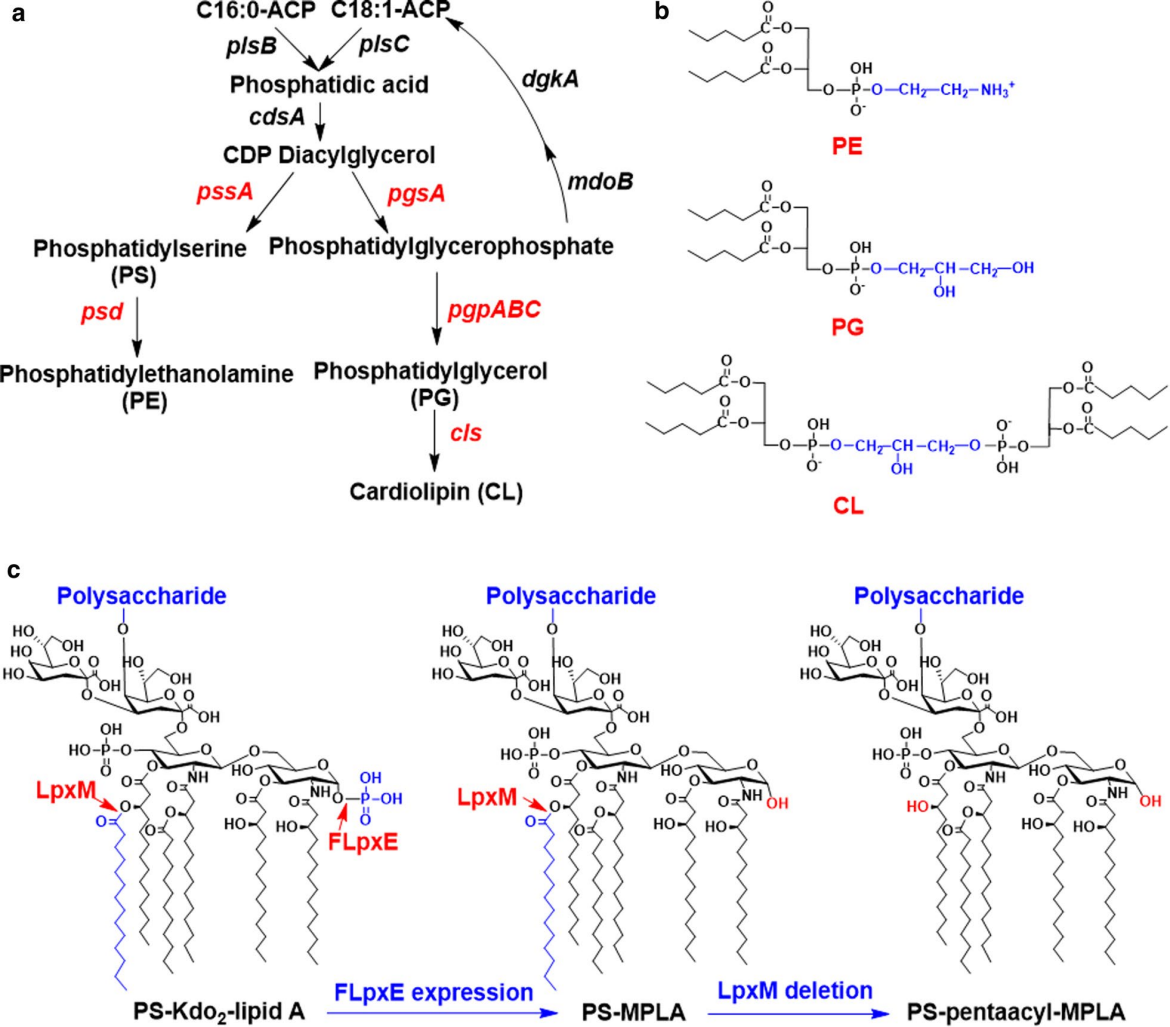
Biosynthesis and structures of lipids in *E. coli*. **a** The biosynthesis pathway of PE, PG and CL in *E. coli*. The red colored genes are critical for the different biosynthesis pathways for PE, PG and CL. **b** Structures of PE, PG and CL. The blue colored parts of the structural formula highlight the differences of PE, PG and CL. **c** Structures of less toxic LPS for *E. coli*. PS-Kdo_2_-lipid A is the wild-type LPS in *E. coli* K-12, PS-MPLA is the less toxic LPS structure after the modification of LpxE deriving *Francisella novicida* in *E. coli* K-12, PS-pentaacyl-MPLA is the least toxic LPS structure after FLpxE expression and LpxM removal in *E. coli* K-12. The key groups and enzymes are colored in red or blue

In *E. coli*, phospholipids and Kdo_2_-lipid A are cytoskeleton structures of the OM and are conservative (Fig. 3). The biosynthesis of lipids in OM is complex in *E. coli* [31, 32]. The phospholipids are mainly consist of ∼ 5% cardiolipin (CL), 20–25% phosphatidylglycerol (PG), and 70–80% phosphatidylethanolamine (PE) [11, 33]. CL is derived from PG, and two molecules of PG form one CL molecule (Fig. 3a). Both PE and PG contain two fatty acid moieties, and four fatty acid moieties in a CL molecule (Fig. 3b). Kdo_2_-lipid A is structured with two molecules of UDP-N-acetylglucosamine (UDP-GlcNAc), two 3-deoxy-D-manno-octulosonic acid (Kdo) residues, and six fatty acid moieties anchoring LPS to the OM (Fig. 3c) [4, 5]. During membrane synthesis, ∼20 million molecules of fatty acids are synthesized in *E. coli* [34].

Modifications of both PG and PE are well-known in resistance mechanisms, especially in response to aminoglycosides and cationic antimicrobial peptides [35]. Kdo_2_-lipid A, as the bioactive center of LPS, is known to be responsible for the toxic effects of infections, and is recognized by the Toll-like receptor 4 (TLR4)/myeloid differentiation protein 2 (MD-2) complex [12, 36, 37]. Kdo_2_-lipid A also represents a significant obstacle for the effective delivery of numerous antimicrobial agents [12]. Moreover, many studies indicated that there are strong links between biosynthetic pathways of phospholipids and lipid A [10]. The links appear valuable for deep understanding of a balance mechanism of membrane components in *E. coli*.

**Fig. 4:**
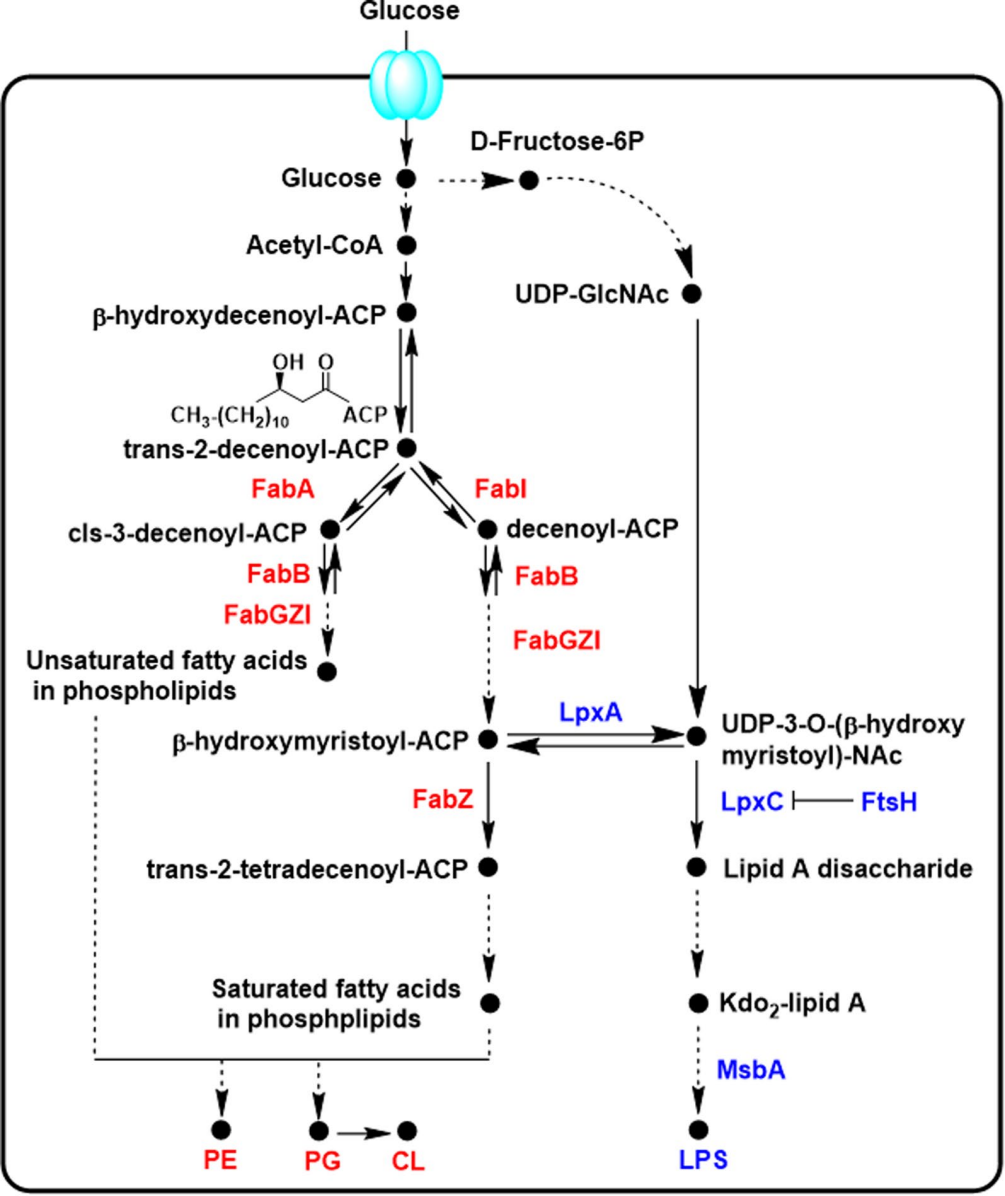
The biosynthesis pathway of the fatty acids chain for *E. coli* LPS and phospholipids from glucose. The enzymes in red are responsible for the biosynthesis of phospholipids, and enzymes in blue are responsible for the biosynthesis of LPS

The fatty acid moieties for phospholipids and lipid A are derived from glucose metabolism, and the pathway is long and complex (Fig. 4) [10, 38]. The modeled interactions between substrates and enzymes under steady-state conditions using Michaelis–Menten and mass action kinetics indicated that the phospholipids and Kdo_2_-lipid A have the common precursor [10]. The glucose uptake happened on the cell membrane, then the most glucose residues flux to Entner–Doudoroff pathway to synthesize Acetyl-CoA. Then two steps of metabolic reactions resulted in the trans-2-decenoyl-ACP, which acts as a common precursor not only fluxing to unsaturated fatty acids with enzymes FabAB, but also fluxing to the saturated fatty acids with enzyme FabI. In *E. coli*, FadR acts as a repressor for the entire set of fad regulon genes [39], and also functions as an activator for unsaturated fatty acid biosynthesis pathway by increasing transcription of both *fabA* and *fabB* [40]. Among the biosynthesis process of saturated fatty acids, the β-hydroxymyristoyl-ACP, as the common precursor, could be shifted to saturated fatty acids by FabZ for phospholipids, also be shifted to lipid A disaccharide by LpxA and LpxC [10]. The balance between phospholipids and LPS biosynthesis are dependent on the balanced LpxA/LpxC and FabZ in *E. coli* cells, and is further regulated by FtsH and Lpp in *E. coli* (Fig. 4). Protease FtsH could regulate the proper amount of LpxC to avoid its overexpression [10]. The Lpp controls the proper pool of free fatty acids for the synthesis of phospholipids and could help to restore the balance with LPS [41]. *E. coli* cells could regulate the composition and amount of phospholipids and Kdo_2_-lipid A as well as the modifications of Kdo_2_-lipid A to better adapt to the various environment conditions.

### ***Engineering phospholipids or Kdo***_***2***_***-lipid A facilitates chemicals production in medicine area***

It is well established that lipids including Kdo_2_-lipid A and phospholipids provide integrity, stability and fluidity to membranes by their 14 to 20 long fatty acids [42]. The shorter fatty acid chains, decreased CL and more unsaturated fatty acids could increase cell fluidity and reduce cell wall rigidity [42, 43], which would benefit the PHA inclusion bodies production [3]. But the depletion of phospholipids including PE, PG and CL resulted in abnormal cell division because phospholipids participate in regulating DnaA protein-mediated initiation of *E. coli* chromosomal replication [44]. Hence, engineering lipids in OM need reasonable design to achieve useful applications in improving *E. coli* robustness and biorenewable tolerance and production [45, 46]. Tan et al. found that increasing the expression of phosphatidylserine synthase (+ *pssA*) could significantly increase the tolerance and production of octanoic acid [45]; also make significant changes for membrane features such as larger bilayer thickness, increased membrane integrity, decreased hydrophobicity and more fatty acids production [46] (Table 1). The expression of *cis*–*trans* isomerase (Cti) from *Pseudomonas aeruginosa* in *E. coli* MG1655 did not increase membrane integrity, but significantly reduce the membrane fluidity. The similar strategy in *Mannheimia succiniciproducens* also facilitates producing trans-unsaturated fatty acid (TUFA) and reinforcing the cell membrane with decreased fluidity, and further improving succinic acid production in *Mannheimia succiniciproducens* [47] (Table 1). Engineering the membrane synthesis pathway by simultaneous overexpression of *plsB* and *plsC* could improve β-carotene production, which is attributed to the increased amount of membrane structures to provide more space to store β-carotene [48] (Table 1).

Kdo_2_-lipid A is the essential component of LPS in *E. coli* and the minimal structural component to sustain bacterial viability [12]. Kdo_2_-lipid A differs from a typical phospholipid by owning six saturated fatty acid chains rather than two saturated or unsaturated chains (Fig. 3). Lipid A disaccharide could be sequentially converted into disaccharide-1-P, Kdo_2_-lipid A by a multiplicity of enzymes. The proper modification of lipid A could decrease the LPS toxicity. The expression of gene *lpxE* derived from *Francisella novicida* in *E. coli* could remove the 1-phosphate group to obtain less toxic PS-monophosphoryl-lipid A (MPLA), and further deleting gene *lpxM* could remove the 3′-secondery acyl chain (C14) to obtain PS-pentaacyl-MPLA with further reduced toxicity (Fig. 3c) [36]. The MPLA had been developed as adjuvant [37], and the PS-MPLA showed decreased ability to activate the TLR4/MD-2 receptor of HKE-Blue hTLR4. Therefore, the attenuated modifications of lipid A in *E. coli* might be helpful to improve application safety for microbial cell factories; and better understanding phospholipids and Kdo_2_-lipid A contributes to design metabolic engineering strategies to improve robustness and security of bacterial producers.

## Polysaccharides of LPS consume lots of nutrients and influence cell phenotypes

**Fig. 5:**
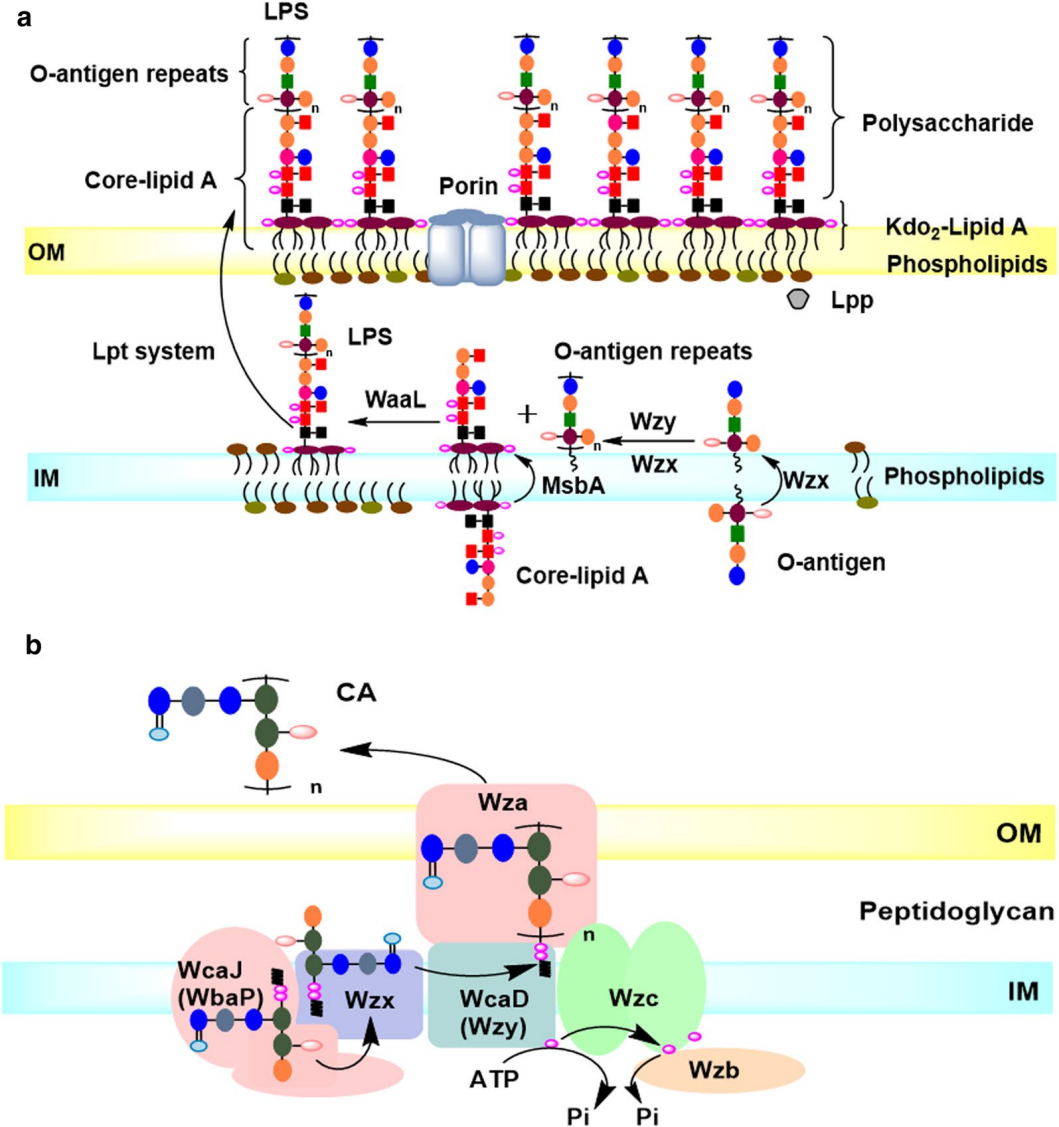
The model of the LPS and CA biosynthesis and transport pathway in *E. coli* K-12. **a** LPS biosynthesis and transport. Core-lipid A is upturned by the transmembrane protein MsbA on the inner membrane, the O-antigen unit is upturned and polymerized by Wzx and Wzy, respectively, forming O-antigen repeats, which are linked to the core-lipid A by WaaL to form the complete LPS, and then is transported into the OM by Lpt system. **b** CA biosynthesis and transport. The CA unit is synthesized in cytoplasmic, and assembled in membrane. Briefly, CA is the repeating polysaccharide unit assembled by various Glycosyltransferases (GT’s) at the C55-lipid linker by Wzx/Wzy dependent pathway, then is translocated toward the periplasm by Wzx flippase; and the polymerization for CA occurs via Wzy polymerase and the polysaccharide co-polymerase protein. The polymerized CA is secreted into the OM by Wza

The LPS structure and biosynthesis pathway have been studied in detail [7]. Briefly, core-lipid A is flipped by the transmembrane protein MsbA on the inner membrane, the O-antigen unit is flipped and polymerized by Wzx and Wzy, respectively, forming O-antigen repeats, which are linked to the core-lipid A by WaaL to form the complete LPS, and then is transported into the OM by Lpt system (Fig. 5a). The polysaccharide chain faces extracellular. The polysaccharide portion includes core oligosaccharide and O-antigen repeats. The LPS polysaccharides are non-essential under normal conditions, but help bacteria resist antibiotics and environmental stresses [34]. In *E. coli* K-12, O-antigen repeats are not present due to the inactivation of *wbbL*, which is responsible for the l-Rhamnose addition to the O-antigen unit. However, the genes involved in the biosynthesis and transfer of O-antigen are still exist in the genome, and sill responsible for the biosynthesis of other EPSs. Therefore, the existing enzymes of O-antigen still lead to the consumption of nutrients.

**Fig. 6:**
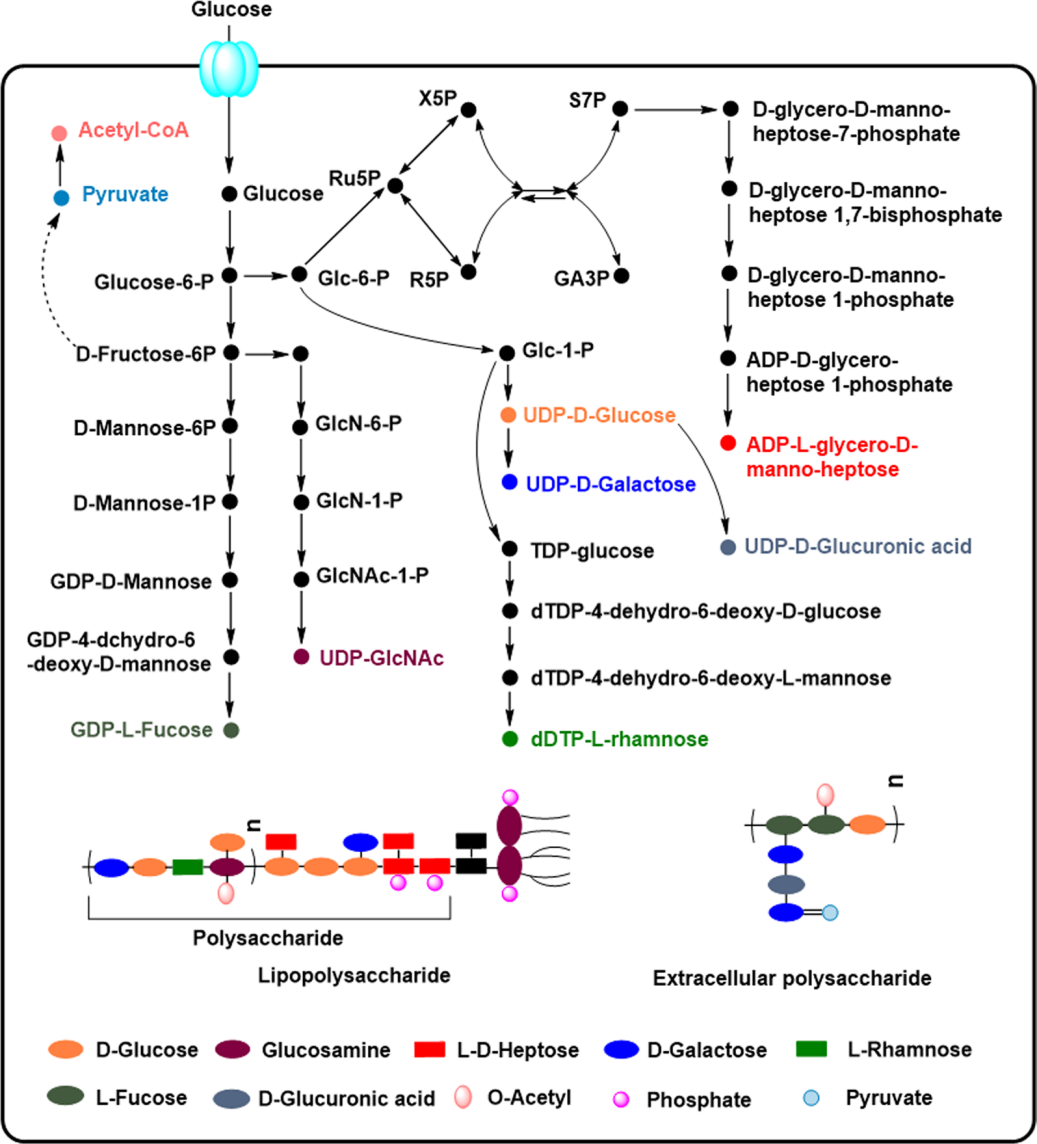
The overview of metabolism pathways from glucose to the sugar nucleotide precursors for LPS and CA polysaccharides biosynthesis. The precursors in different colors represents the sugar components shown with the same color in LPS or CA structure. Glc-6-P, Glucose-6-phosphate; Glc-1-P: Glucose-1-phosphate; Ru5P: Ribulose-5-phosphate; X5P: xylulose-5-phosphate; R5P: Ribose-5-phosphate; S7P: sedoheptulose-7-phosphate; GlcN-6-P: 6-phospho-gluconate; GlcN-1-P: 1-phospho-gluconate; GlcNAc-1-P: Glucosamine-1-phosphate. The metabolites in different colors are corresponding with the differently colored groups in LPS and CA molecules

**Fig. 7:**
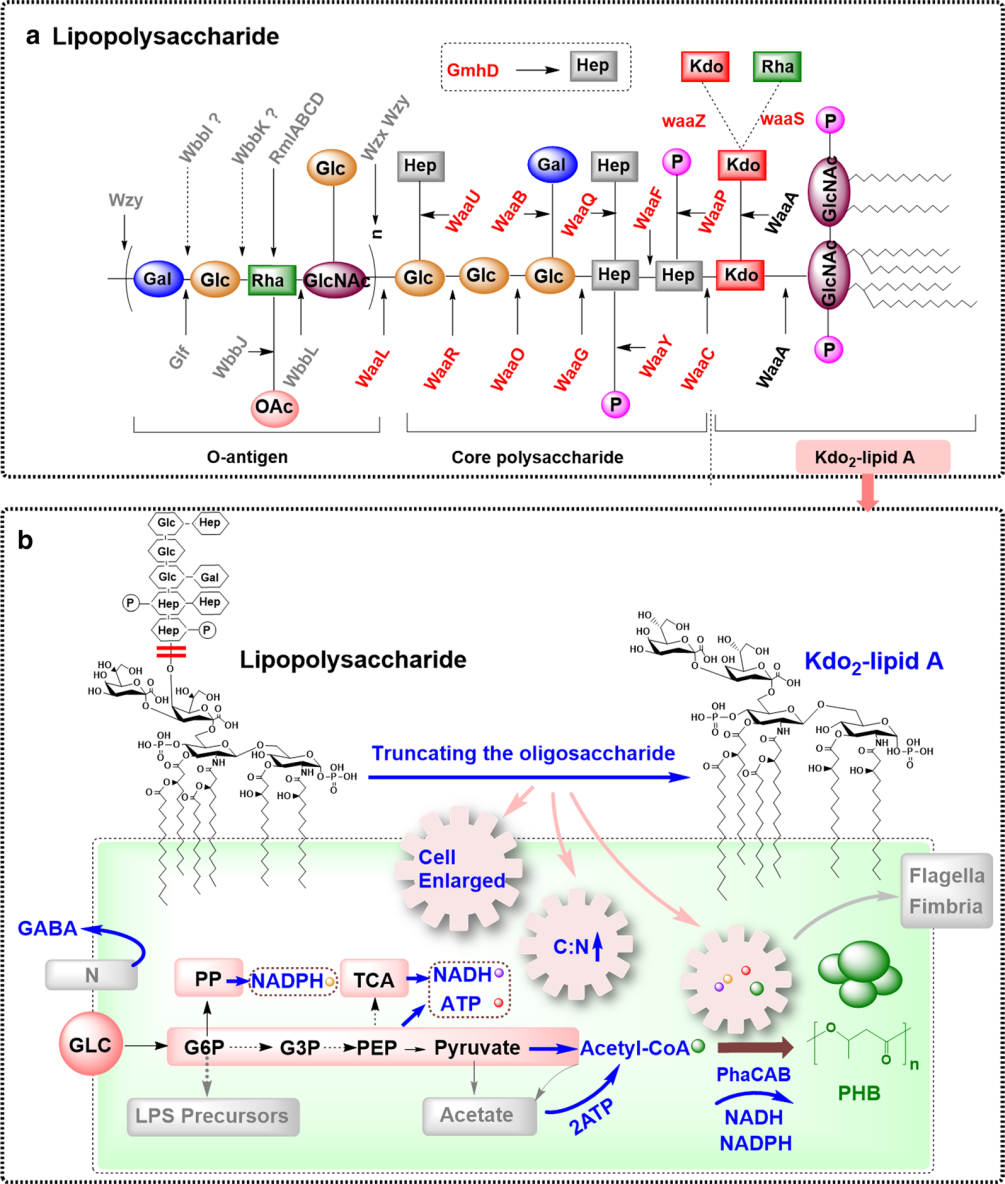
Truncating LPS can efficiently improve the PHB production in *E. coli*. **a** The structure of LPS and the genes relevant to the polysaccharides portion of LPS. The enzymes in red are encoded by core cluster, and enzymes in gray are encoded by O-antigen cluster. **b** The mechanism analysis for the improved PHB accumulation by truncating LPS in *E. coli*. EMP: Entner–Doudoroff pathway; TCA: Tricarboxylic acid cycle; PP: Pentose Phosphate; GLC: Glucose; G6P: Glucose-6-phosphate; 3PG: 3-phosphoglycerate; PEP: phosphoenol pyruvate; PHB: poly-3-hydroxybutyrate. The key factors are highlighted with blue color for improving PHB production

The core of LPS is complete and present in *E. coli* K-12. The core is a branched oligosaccharide chain with phosphoryl substituents [49], and the neighboring LPS core molecules could be bridged by negatively charged phosphoryl substituents through divalent cation interactions [49]. In addition to the Kdo residue, the general sugar groups found in the core oligosaccharides are four l-glycero-d-manno-heptose (Hep), three d-glucose and one d-galactose (Fig. 5a) [7], they are most derived from the carbon source glucose, and the biosynthesis pathway of their precursors is summarized in the Fig. 6. The genes responsible for core exist in one cluster (Fig. 2 and Fig. 7) [50]. GmhD-WaaFC and WaaQ are required for biosynthesis and transfer of l, d-heptose in the inner core [49]. WaaP and WaaY are responsible for the phosphate modifications of the inner core. The “ligase” WaaL coded by *waaL* is required to link O-polysaccharide to the completed core [51, 52]. WaaG, WaaB, WaaO, WaaR and WaaU are required for the addition of d-glucose, d-galactose and l, d-heptose in the outer core [7]. WaaS and WaaZ may be responsible for the addition of l-Rhamnose and Kdo on the second Kdo under certain conditions, but are non-essential. While the WaaA responsible for the bifunctional Kdo transferase [53] and a “non-LPS” enzyme CoaD encoding phosphopantetheine adenylyltransferase are essential [54]. Importantly, the knockout of *waaCF* encoding heptosyltransferase or *gmhD* encoding ADP-l-glycero-d-manno-heptose-6-epimerase could block the polysaccharide portion attachment to Kdo_2_-lipid A. Heptosyltransferase adds an l-d-heptose to Kdo_2_-lipid A, forming Hep-Kdo_2_-lipid A and Hep_2_-Kdo_2_-lipid A; and ADP-l-*glycero*-d-*manno*-heptose-6-epimerase coverts the donor of ADP-l-d-heptose from ADP-D-D-heptose. *E. coli* K-12 W3110 mutants WBB06 [55] and WJW00 [56] could be used to produce Kdo_2_-lipid A. Furthermore, the least toxic structure Kdo_2_-P-MPLA could be synthesized by the mutant HWB02 [37].

### Truncating the polysaccharides portion of LPS benefits to optimize the features and efficiency of microbial cell factories

In *E. coli*, the blocks to different groups of the polysaccharide portion of LPS could cause different influences on cell phenotypes (Table 2). We speculated that certain characteristics observed in some mutants could be applied in industrial fermentation [57]. The deeply truncated LPS had strongest impacts on *E. coli* cells, mainly including much lower toxicity, lack of motility, significantly increased OM permeability, much stronger auto-aggregation ability, sharply dropped biofilm formation ability and decreased antibiotic resistance, compared to the wild-type *E. coli* control (Table 2). The LPS mutants could be attenuated and may be more suitable to apply in industrial applications. The lack of motility together with the LPS truncation benefit saving more resources and energy sources for cells. The stronger auto-aggregation ability could reduce the costs for cells collection and cells broken. The weaker biofilm formation could benefit the sterilization of fermenters and contribute to avoid bacterial contamination.

**Table 2:** Effects on *E. coli* cell by LPS structure modification

Gene mutation	LPS component	Effects on cell	Refs.
Deletion of *waaB*	Glc_3_- Hep_3_-Kdo_2_-lipid A	Auto-aggregation (+)	[117]
Deletion of *waaO* or *waaR*	Glc-Hep_3_-Kdo_2_-lipid A or Gal-Glc_2_- Hep_3_-Kdo_2_-lipid A	Biofilm formation (−), auto-aggregation (+)	[117]
Deletion of *waaC*, *waaF*, *waaP* or *waaG*	Kdo_2_-lipid A, Hep-Kdo_2_-lipid A, or Hep_3_-Kdo_2_-lipid A	Flagella (−), biofilm formation (−), auto-aggregation (+),antibiotic resistance (−)	[117, 118]
Deletion of *gmhD* or *waaC*	Kdo_2_-lipid A	OM permeability (+), auto-aggregation (+), biofilm formation (−), antibiotic resistance (−),	[56, 117]
Deletion of *waaCF* and *lpxM*, integration of F*lpxE*	Kdo_2_-pentaacyl-MPLA	OM permeability (+), auto-aggregation (+), stimulating activities (−), antibiotic resistance (−)	[37]

“ + ”: increased, “-”: decreased

It is worth paying attention to the potential advantage of saving carbon and energy sources in LPS mutants. In wild-type *E. coli* K-12, the biosynthesis of LPS core consumes approximately 16 × 10^6^ more molecules of sugar than the minimal structure Kdo_2_-lipid A for each cell. When the LPS is truncated, the saved carbon sources may be fluxed to the central metabolic pathway to further produce more target products (Table 1) In *Pseudomonas putida*, the production of poly-3-hydroxybutyrate (PHB) decreases with the increase of LPS synthesis [58]; and PHB could be elevated to 38%wt in a LPS mutant *Ralstonia eutropha* DO10, much higher than the control H16 (25%wt) [59]; moreover, Zhang et al. found that weakening cell membrane and reducing cell envelope rigidity could significantly enhance the PHB production in *E. coli* JM109 [3]; and we recently demonstrated that LPS truncation can efficiently improve PHB production (Fig. 7) [2]. The truncation of LPS facilitates an increase of 75.6–200% in different *E. coli* strains [2]. These metabolic changes indicated that the lack of polysaccharide portion of LPS had significant influences on cells, which provides important theoretical reference for engineering better microbial cell factories for many products, especially for PHA inclusion bodies.

In addition, we also found that several deletion mutants for the polysaccharide of LPS could efficiently improve the colanic acid (CA) production [60]. The mutant lacking WaaL-WaaQ in MG1655 significantly improved CA production by 5.6-fold to 0.278 g/ L than that of MG1655; and mutant lacking WaaF could also facilitate CA accumulation. LPS pathway might have a huge priority to CA pathway to use the common precursors. The results indicated that the reasonable engineering of the polysaccharide portion of LPS could applied in improving CA production.

### Biosynthesis of EPSs consumes lots of nutrients and leads to more biofilms

The EPSs are secreted or covalently linked to the outer layer LPS of OM. The EPS contains nucleic acids, lipids, and proteins in addition to polysaccharides, and takes up to 90% of the dry weight of the biofilm [61]. Functions of polysaccharides of EPS in biofilms formation is concluded in Table 3 [61]. In *E. coli* K-12, the EPS is mainly formed as CA, which contributes to the resistance of bacteria to environmental stresses (desiccation, heat, acid, osmotic and oxidative stresses) [62]. CA is critical for the formation of the complex three-dimensional structure and depth of *E. coli* biofilms [63]. CA expression is transcriptionally regulated by the RcsC-YojN-RcsB phosphorelay system [64] and is assembled by a Wzy-dependent polymerization system (Fig. 5b) [65]. The involved 21 genes are distributed the operon near the chromosomal wb* (O-antigen biosynthesis) genes (Fig. 2) [66]. In overview, undecaprenol diphosphate (und-PP)-linked oligosaccharides unit are formed by glycosyltransferase Wca*, and then flipped across the inner membrane by Wzx protein and polymerized by WcaD (Wzy), and are exported to the surface at last [64]. One CA unit is composed of two molecules of l-fucose, two molecules of d-galactose, one molecule of d-glucuronic acid, one molecule of d-glucose, one O-acetyl linked to the middle l-fucose and one pyruvate linked acetalically to galactose (Fig. 5b) [62, 66]. These sugar nucleotide precursors for CA could be synthesized from glucose (Fig. 6). The common sugar nucleotide precursors for CA and LPS polysaccharide are UDP-d-glucose, UDP-l-galactose, dDTP-l-rhamnose and O-Acetyl. Thus, when the LPS polysaccharide biosynthesis is blocked, these precursors might flux to the CA biosynthesis.

**Table 3:** Functions of polysaccharides in biofilms formation [61]

EPS function	Effect on biofilm
Adhesion	Allows the initial steps and the long-term attachment of whole biofilms to surfaces
Aggregation of bacterial cells	Enables bridging between cells and cell–cell recognition
Cohesion of biofilms	Determines biofilm architecture and allows cell–cell communication
Protective barrier	Confers resistance to nonspecific and specific host defense
Sink for excess energy	Stores excess carbon under unbalanced carbon to nitrogen ratios

The synthesis of CA also consumes a lot of carbon sources. Under some stress conditions, when most of the cells are killed, many of those colonies that survive may be mucoid [67]. In this way, CA has become the main component of the biofilm in *E. coli* K-12 [63]. CA production is not required for surface attachment, however, CA is critical for the formation of the complex three-dimensional structure and depth of *E. coli* biofilms [63]. Bacterial biofilms have been described as sessile bacterial communities that live attached to each other and stick onto surfaces [68, 69]. Biofilm is so abundant in natural environments, but can also cause persistent, antibiotic-resistant infections [68, 70], especially impinge significantly upon our industrialized world [71, 72]. This is a trouble for industrial fermentation due to the possibility of clogging pipes [73, 74], contamination [75], insecurity [76, 77] and biofouling [78, 79]. Excessive growth of biofilms and associated EPS would also greatly limit the diffusion of substrates and nutrients to the cells [80]. Therefore, metabolic engineering CA might be used as a tool to control biofilm formation. Blocking or reducing the CA formation on the cell surface by deleting genes relevant to CA biosynthesis or its regulatory genes rcs* could not only save nutrients but also significantly reduce biofilm formation [66].

### Flagella and fimbria consume lots of energy sources

In addition to the lipids and polysaccharides, there are many flagella and fimbriae observed on the cell surface in *E. coli* K-12. The abundant flagella and type I fimbriae distribute on the surface of the bacterium (Fig. 1) [81]. The production and the rotation of flagella and pili are energy-demanding processes for the cell. In *E. coli*, one flagella filament consists of about 20,000 FliC protein, up to 10 μm and 20 nm in diameter [82]. Previous research has established that a flagellum is assembled from the inside out, beginning with the basal body embedded in the cell membrane [83]. The *E. coli* flagellum consists of six components: a basal body (including MS ring, P ring, and L ring), a reversible rotary motor, a switch, a short proximal hook, a long helical filament, and a type III flagellar secretion system (T3FSS) as the export apparatus (Fig. 1) [83–85]. The involving genes are distributed three clusters (Fig. 2), [16]. Using the transmembrane electrochemical ion motive force to power the bacterial flagellar motor, fast rotating flagella can propel the cell body at a speed of 15–100 µm/s [85–88]. FlhD and FlhC are flagellum-specific transcriptional activators responsible for the expression of all other flagellar genes [89]. The flagellum-specific factor, FliA (σ^28^) together with the FlgM (anti-σ^28^) regulates the expression of level III genes to finish the complete biosynthesis of flagella [90]. Level III includes genes for late morphogenesis, energy transduction, and signal processing [91]. The flagellar filament is assembled from tens of thousands of flagellin subunits that are exported through the flagellar type III secretion system [92]. The export efficiency of the flagellar type III secretion system depends on its energy source [93]. Insufficient cytoplasmic flagellin supply results in the pauses in flagellar growth, thus different flagella on the same *E. coli* cell show variable growth rates with correlation [92]. According to this, the biosynthesis and rotor of numerous flagella in *E. coli* consume lots of energy, and removing structural proteins including rotary motor or the regulators controlling flagella rotor may benefit to save energy.

*E. coli* cells exhibit many type I fimbriae are filamentous structures on their surfaces [94]. Type I fimbriae are filamentous tubular structures of 0.2–2.0 mm in length and 5–7 nm in diameter, with between 500 and 3000 major FimA pilins forming the fimbrial shaft [94, 95], monitoring in the OM. These fimbriae are secreted by the chaperone/usher pathway [96]. The fimbriae biosynthesis involves 9 genes in one cluster (Fig. 2) with *fimA-H* operon located near the regulatory recombinase *fimB* and *fimE* transcription units[97–99]. These gene mutants exhibited strongly repressed swarming motilities but no significant repression of swimming motility [100]. The *fimA*, *fimC*, and *fimD* gene products are essential for constructing the fimbrial fiber. The *fimF* and *fimH* mutants were reported to have markedly reduced numbers of fimbriae per cell [100]. Type I fimbriae contribute to bladder colonization by binding to α-d-mannosylated proteins using a tiplocated FimH adhesin in *E. coli* [94, 101]. Cpx-signaling system consisting of CpxA (sensor kinase) and CpxR (response regulator) pathway is involved in the regulation of adhesion-induced gene expression, deletion of *cpxA* could completely inhibit the swarming in *E. coli*, but not in *cpxR* mutant [100]. In addition, the energy coupled with TonB for the secretion is generated at the IM by the proton motive force, which facilitates the gating in the OM channel [102]. Powering the large conformational rotation of the plug domain also need a lot of energy sources [96]. Therefore, the type I fimbria in *E. coli* consumes lots of energy, and could be removed by deleting structural genes or signaling gene *cpxA*.

### Removal of flagella and fimbria benefits to reduce biofilm and to increase growth rate, energy, co-factors, and PHA production

Previous studies proved that cell-surface polymers such as filamentous proteins fimbriae and flagella could influence the bacterial attachment process on hosts or inert surfaces [103]. Thus the flagella and fimbriae can stabilize the biofilm matrix and colonization on infected hosts [61]. In *E. coli* K-12, motility is important for initiating biofilm formation at least at the early stages [104], and lack of motility significantly reduced the biofilm formation [105]. Deletion of *flhD* or *fliC* could result in flagella structure defect and a severe biofilm defect [104]. Likewise, mutations that paralyzed the flagellar motor but left the intact structure could also cripple the biofilm formation [104]. Type I fimbriae largely increased the attachment and the transformation ratio during the first phase of biofilm formation [106].

Thus, the existence of flagella and pili is also harmful to the bacteria industrial fermentation. In addition, the production and the rotation of flagella and pili are energy-demanding processes and a considerable metabolic burden for cells. In order to maintain the motility, cells consume more ATP [82, 94]. In *E. coli*, flagellar synthesis imposes a cost of approximately 2% of the biosynthetic energy expenditure of the cell [18, 107]. Thus, paralyzing the flagella or pili biosynthesis could be a good metabolic engineering strategy to save more resources and energy source. Indeed, the removal of entire flagella and fimbriae biosynthesis gene clusters had been performed for the minimal genome platform constructions in *E. coli* [108–114] and *P. putida* [18, 115]. Although flagella are important structures for coping with environmental circumstances, under certain conditions, not having this organelle could provide the bacteria with more energy and/or reducing power. But the expression of flagella is not crucial for survival, and losing this appendage could save the imposed metabolic burden for cells [18, 19, 110, 113–115]. Especially in *P. putida*, non-flagellated mutant could increase biomass, increase 30% energy charge and 20% NADPH/NADP^+^ ratio, improve tolerance to oxidative stress and stationary phase viability compared with the wild-type KT2440 [18, 115]. Our recently study also showed that deletion of 76 genes involved in flagella and pili in *P. putida* significantly reduced the biofilm formation and intracellular level of c-di-GMP, but grew faster, and significantly enhanced the PHA production [116]. We found that the biomass, PHA yield, and content of deletion mutant WJPP03 increased 19.1, 73.4, and 45.6%, respectively [116]. Therefore, deletion of the genes involved in flagella and fimbriae biosynthesis could be applied in the metabolic engineering constructions for robust microbial cell factory.

## Conclusion

*Escherichia coli* K-12, as a typical Gram-negative bacteria, has been widely used as a cell factory producer for many products, such as amino acids, organic acids and PHA inclusion bodies, especially applications in food, medicine or renewable biological resources. However, *E. coli* still has the food insecurity due to the presence of some molecules in OM, because Kdo_2_-lipid A could cause immune responses, polysaccharide of LPS, EPSs, flagella and fimbria could cause biofilm formation, which leads to bacterial contamination. In this review, we concluded the structures, biosynthesis, function, influences and metabolic engineering applications of the OM molecules in *E. coli*, and try to provide some references for constructing better microbial cell factories.

The biological safety of gram-negative bacteria for industrial application attracts our attention. According to the analysis for the OM molecules, We suggested that efficient strategies targeting membrane engineering could be developed for common bacterial producers. To reduce LPS toxicity, the Kdo_2_-lipid A portion of LPS could be modified to the structure PS-pentaacyl-MPLA by expressing FLpxE expression and removing LpxM in *E. coli* K-12. In addition to Kdo_2_-lipid A, the polysaccharide portion of LPS could be truncated to increase the permeability and auto-aggregation, and save more carbon sources, and decrease the biofilm formation [56]. The EPSs could also be removed to avoid consuming many carbon sources and energy, and reduce biofilm formation. The flagella and fimbria are also usually deleted for the construction of a minimal genome strain platform [6, 19, 114], we also suggested that flagella and fimbria should be removed in common metabolic engineering to yield genetically defined overproducers or starting platforms. It is believed that proper membrane engineering would not only reduce the cell burden, but also improve the bacterial fermentation features for constructing better synthetic biological platforms. In conclusion, the systematical and comprehensive understanding of the OM molecules would benefit developing more potential metabolic engineering strategies to improve the efficiency and safety of microbial cell factories.

## Data Availability

Not applicable.

## References

[1] Guo L, Diao W, Gao C, Hu G, Ding Q, Ye C, Chen X, Liu J, Liu L (2020). Engineering *Escherichia coli* lifespan for enhancing chemical production. Nature Catalysis.

[2] Wang J, Ma W, Fang Y, Zhang H, Liang H, Li Y, Wang X (2020). Truncating the structure of lipopolysaccharide in *Escherichia coli* can effectively improve poly-3-hydroxybutyrate production. ACS Synth Biol.

[3] Zhang XC, Guo Y, Liu X, Chen XG, Wu Q, Chen CQ (2018). Engineering cell wall synthesis mechanism for enhanced PHB accumulation in *E. coli*. Metab Eng.

[4] Wang X, Quinn PJ (2010). Lipopolysaccharide: biosynthetic pathway and structure modification. Prog Lipid Res.

[5] Ruiz N, Kahne D, Silhavy TJ (2009). Transport of lipopolysaccharide across the cell envelope: the long road of discovery. Nat Rev Microbiol.

[6] Iwadate Y, Honda H, Sato H, Hashimoto M, Kato J (2011). Oxidative stress sensitivity of engineered *Escherichia coli* cells with a reduced genome. FEMS Microbiol Lett.

[7] Raetz CR, Whitfield C (2002). Lipopolysaccharide endotoxins. Annu Rev Biochem.

[8] Nikaido H (2003). Molecular basis of bacterial outer membrane permeability revisited. Microbiol Mol Biol Rev.

[9] Koebnik R, Locher KP, Van Gelder P (2000). Structure and function of bacterial outer membrane proteins: barrels in a nutshell. Mol Microbiol.

[10] Emiola A, Andrews SS, Heller C, George J (2016). Crosstalk between the lipopolysaccharide and phospholipid pathways during outer membrane biogenesis in *Escherichia coli*. Proc Natl Acad Sci USA.

[11] Renner LD, Weibel DB (2011). Cardiolipin microdomains localize to negatively curved regions of *Escherichia coli* membranes. Proc Natl Acad Sci USA.

[12] Wang X, Quinn PJ, Yan A (2015). Kdo_2_ -lipid A: structural diversity and impact on immunopharmacology. Biol Rev Camb Philos Soc.

[13] Nickerson NN, Mainprize IL, Hampton L, Jones ML, Naismith JH, Whitfield C (2014). Trapped translocation intermediates establish the route for export of capsular polysaccharides across *Escherichia coli* outer membranes. Proc Natl Acad Sci USA.

[14] Miller SI, Salama NR (2018). The gram-negative bacterial periplasm: size matters. PLoS Biol.

[15] Patel TN, Park AH, Banta S (2014). Genetic manipulation of outer membrane permeability: generating porous heterogeneous catalyst analogs in *Escherichia coli*. ACS Synth Biol.

[16] Liu R, Ochman H (2007). Stepwise formation of the bacterial flagellar system. Proc Natl Acad Sci USA.

[17] Chen YW, Teng CH, Ho YH, Jessica Ho TY, Huang WC, Hashimoto M, Chiang IY, Chen CS (2014). Identification of bacterial factors involved in type 1 fimbria expression using an *Escherichia coli* K12 proteome chip. Mol Cell Proteomics.

[18] Martinez-Garcia E, Nikel PI, Chavarria M, de Lorenzo V (2014). The metabolic cost of flagellar motion in *Pseudomonas putida* KT2440. Environ Microbiol.

[19] Posfai G, Plunkett G, Feher T, Frisch D, Keil GM, Umenhoffer K, Kolisnychenko V, Stahl B, Sharma SS, de Arruda M (2006). Emergent properties of reduced-genome *Escherichia coli*. Science.

[20] Wood TK (2009). Insights on *Escherichia coli* biofilm formation and inhibition from whole-transcriptome profiling. Environ Microbiol.

[21] Delcour AH (2009). Outer membrane permeability and antibiotic resistance. Biochim Biophys Acta.

[22] Kaeriyama M, Machida K, Kitakaze A, Wang H, Lao Q, Fukamachi T, Saito H, Kobayashi H (2006). OmpC and OmpF are required for growth under hyperosmotic stress above pH 8 in *Escherichia coli*. Lett Appl Microbiol.

[23] Patel DS, Re S, Wu EL, Qi Y, Klebba PE, Widmalm G, Yeom MS, Sugita Y, Im W (2016). Dynamics and interactions of OmpF and LPS: influence on pore accessibility and ion permeability. Biophys J.

[24] Bekhit A, Fukamachi T, Saito H, Kobayashi H (2011). The role of OmpC and OmpF in acidic resistance in *Escherichia coli*. Biol Pharm Bull.

[25] Park JS, Lee WC, Yeo KJ, Ryu KS, Kumarasiri M, Hesek D, Lee M, Mobashery S, Song JH, Kim SI (2012). Mechanism of anchoring of OmpA protein to the cell wall peptidoglycan of the gram-negative bacterial outer membrane. FASEB J.

[26] Samsudin F, Boags A, Piggot TJ, Khalid S (2017). Braun's Lipoprotein facilitates OmpA interaction with the *Escherichia coli* cell wall. Biophys J.

[27] Boags AT, Samsudin F, Khalid S (2019). Binding from both sides: TolR and full-length OmpA bind and maintain the local structure of the *E. coli* cell wall. Structure.

[28] Höltje JV (1998). Growth of the stress-bearing and shape-maintaining murein sacculus of *Escherichia coli*. Microbiol Mol Biol Rev.

[29] Smith SG, Mahon V, Lambert MA, Fagan RP (2007). A molecular Swiss army knife: OmpA structure, function and expression. FEMS Microbiol Lett.

[30] Wang Y (2002). The function of OmpA in *Escherichia coli*. Biochem Biophys Res Commun.

[31] Shin J, Yu J, Park M, Kim C, Kim H, Park Y, Ban C, Seydametova E, Song YH, Shin CS (2019). Endocytosing *Escherichia coli* as a whole-cell biocatalyst of fatty acids. ACS Synth Biol.

[32] Wu T, Li S, Ye L, Zhao D, Fan F, Li Q, Zhang B, Bi C, Zhang X (2019). Engineering an artificial membrane vesicle trafficking system (AMVTS) for the excretion of beta-carotene in *Escherichia coli*. ACS Synth Biol.

[33] Tan BK, Bogdanov M, Zhao J, Dowhan W, Raetz CR, Guan Z (2012). Discovery of a cardiolipin synthase utilizing phosphatidylethanolamine and phosphatidylglycerol as substrates. Proc Natl Acad Sci USA.

[34] Raetz CR, Guan Z, Ingram BO, Six DA, Song F, Wang X, Zhao J (2009). Discovery of new biosynthetic pathways: the lipid A story. J Lipid Res.

[35] Band VI, Weiss DS (2015). Mechanisms of antimicrobial peptide resistance in gram-negative bacteria. Antibiotics.

[36] Han Y, Li Y, Chen J, Tan Y, Guan F, Wang X (2013). Construction of monophosphoryl lipid A producing *Escherichia coli* mutants and comparison of immuno-stimulatory activities of their lipopolysaccharides. Mar Drugs.

[37] Wang B, Han Y, Li Y, Li Y, Wang X (2015). Immuno-stimulatory activity of *Escherichia coli* mutants producing Kdo2-monophosphoryl-lipid A or Kdo2-pentaacyl-monophosphoryl-lipid A. PLoS ONE.

[38] Kenanov D, Kaleta C, Petzold A, Hoischen C, Diekmann S, Siddiqui RA, Schuster S (2010). Theoretical study of lipid biosynthesis in wild-type *Escherichia coli* and in a protoplast-type L-form using elementary flux mode analysis. FEBS J.

[39] Henry MF, Cronan JE (1991). *Escherichia coli* transcription factor that both activates fatty acid synthesis and represses fatty acid degradation. J Mol Biol.

[40] Campbell JW, Cronan JE (2001). Escherichia coli FadR positively regulates transcription of the *fabB* fatty acid biosynthetic gene. J Bacteriol.

[41] Klein G, Raina S (2015). Regulated control of the assembly and diversity of LPS by noncoding sRNAs. Biomed Res Int.

[42] Mihoub M, El May A, Aloui A, Chatti A, Landoulsi A (2012). Effects of static magnetic fields on growth and membrane lipid composition of *Salmonella typhimurium* wild-type and dam mutant strains. Int J Food Microbiol.

[43] Romantsov T, Guan Z, Wood JM (2009). Cardiolipin and the osmotic stress responses of bacteria. Biochim Biophys Acta.

[44] Saxena R, Fingland N, Patil D, Sharma AK, Crooke E (2013). Crosstalk between DnaA protein, the initiator of *Escherichia coli* chromosomal replication, and acidic phospholipids present in bacterial membranes. Int J Mol Sci.

[45] Tan Z, Khakbaz P, Chen Y, Lombardo J, Yoon JM, Shanks JV, Klauda JB, Jarboe LR (2017). Engineering *Escherichia coli* membrane phospholipid head distribution improves tolerance and production of biorenewables. Metab Eng.

[46] Tan Z, Yoon JM, Nielsen DR, Shanks JV, Jarboe LR (2016). Membrane engineering via trans unsaturated fatty acids production improves *Escherichia coli* robustness and production of biorenewables. Metab Eng.

[47] Ahn JH, Lee JA, Bang J, Lee SY (2018). Membrane engineering via trans-unsaturated fatty acids production improves succinic acid production in *Mannheimia succiniciproducens*. J Ind Microbiol Biotechnol.

[48] Wu T, Ye L, Zhao D, Li S, Li Q, Zhang B, Bi C, Zhang X (2017). Membrane engineering-A novel strategy to enhance the production and accumulation of beta-carotene in *Escherichia coli*. Metab Eng.

[49] Yethon JA, Heinrichs DE, Monteiro MA, Perry MB, Whitfield C (1998). Involvement of *waaY*, *waaQ*, and *waaP* in the modification of *Escherichia coli* lipopolysaccharide and their role in the formation of a stable outer membrane. J Biol Chem.

[50] Schnaitman CA, Klena JD (1993). Genetics of lipopolysaccharide biosynthesis in *enteric bacteria*. Microbiol Rev.

[51] Whitfield C, Amor PA, Koplin R (1997). Modulation of the surface architecture of gram-negative bacteria by the action of surface polymer:lipid A-core ligase and by determinants of polymer chain length. Mol Microbiol.

[52] Heinrichs DE, Monteiro MA, Perry MB, Whitfield C (1998). The assembly system for the lipopolysaccharide R2 core-type of *Escherichia coli* is a hybrid of those found in *Escherichia coli* K-12 and *Salmonella enterica*. Structure and function of the R2 WaaK and WaaL homologs. J Biol Chem.

[53] Clementz T, Raetz CR (1991). A gene coding for 3-deoxy-d-manno-octulosonic-acid transferase in *Escherichia coli*. Identification, mapping, cloning, and sequencing. J Biol Chem.

[54] Geerlof A, Lewendon A, Shaw WV (1999). Purification and characterization of phosphopantetheine adenylyltransferase from *Escherichia coli*. J Biol Chem.

[55] Brabetz W, Muller-Loennies S, Holst O, Brade H (1997). Deletion of the heptosyltransferase genes *rfaC* and *rfaF* in *Escherichia coli* K-12 results in an Re-type lipopolysaccharide with a high degree of 2-aminoethanol phosphate substitution. Eur J Biochem.

[56] Wang J, Ma W, Wang Z, Li Y, Wang X (2014). Construction and characterization of an *Escherichia coli* mutant producing Kdo(2)-lipid A. Mar Drugs.

[57] Zhao A, Hu X, Wang X (2017). Metabolic engineering of *Escherichia coli* to produce gamma-aminobutyric acid using xylose. Appl Microbiol Biotechnol.

[58] Goff M, Nikodinovic-Runic J, O'Connor KE (2009). Characterization of temperature-sensitive and lipopolysaccharide overproducing transposon mutants of *Pseudomonas putida* CA-3 affected in PHA accumulation. FEMS Microbiol Lett.

[59] Brandt U, Raberg M, Voigt B, Hecker M, Steinbüchel A (2012). Elevated poly(3-hydroxybutyrate) synthesis in mutants of *Ralstonia eutropha H16* defective in lipopolysaccharide biosynthesis. Appl Microbiol Biotechnol.

[60] Wang C, Zhang H, Wang J, Chen S, Wang Z, Zhao L, Wang X (2020). Colanic acid biosynthesis in *Escherichia coli* is dependent on lipopolysaccharide structure and glucose availability. Microbiol Res.

[61] Flemming HC, Wingender J (2010). The biofilm matrix. Nat Rev Microbiol.

[62] Whitfield C, Paiment A (2003). Biosynthesis and assembly of Group 1 capsular polysaccharides in *Escherichia coli* and related extracellular polysaccharides in other bacteria. Carbohydr Res.

[63] Danese PN, Pratt LA, Kolter R (2000). Exopolysaccharide production is required for development of *Escherichia coli* K-12 biofilm architecture. J Bacteriol.

[64] Reid AN, Whitfield C (2005). functional analysis of conserved gene products involved in assembly of *Escherichia coli* capsules and exopolysaccharides: evidence for molecular recognition between Wza and Wzc for colanic acid biosynthesis. J Bacteriol.

[65] Schmid J, Sieber V, Rehm B (2015). Bacterial exopolysaccharides: biosynthesis pathways and engineering strategies. Front Microbiol.

[66] Ren G, Wang Z, Li Y, Hu X, Wang X (2016). Effects of Lipopolysaccharide Core Sugar Deficiency on Colanic Acid Biosynthesis in *Escherichia coli*. J Bacteriol.

[67] Gottesman S, Stout V (1991). Regulation of capsular polysaccharide synthesis in *Escherichia coli* K12. Mol Microbiol.

[68] Costerton JW, Stewart PS, Greenberg EP (1999). Bacterial biofilms: a common cause of persistent infections. Science.

[69] Zhang J, Poh CL (2018). Regulating exopolysaccharide gene wcaF allows control of *Escherichia coli* biofilm formation. Sci Rep.

[70] Goto T, Nakame Y, Nishida M, Ohi Y: Bacterial biofilms and catheters in experimental urinary tract infection. *Int J Antimicrob Agents* 1999, 11:227–231; discussion 237–229. 10.1016/s0924-8579(99)00019-9 10394975

[71] Alvarez-Ordonez A, Coughlan LM, Briandet R, Cotter PD (2019). Biofilms in food processing environments: challenges and opportunities. Annu Rev Food Sci Technol.

[72] Wong AC (1998). Biofilms in food processing environments. J Dairy Sci.

[73] Halan B, Buehler K, Schmid A (2012). Biofilms as living catalysts in continuous chemical syntheses. Trends Biotechnol.

[74] Yoshida K, Tashiro Y, May T, Okabe S (2015). Impacts of hydrophilic colanic acid on bacterial attachment to microfiltration membranes and subsequent membrane biofouling. Water Res.

[75] Galie S, Garcia-Gutierrez C, Miguelez EM, Villar CJ, Lombo F (2018). Biofilms in the food industry: health aspects and control methods. Front Microbiol.

[76] Kim HJ, Oh T, Baek SY (2018). Multidrug resistance, biofilm formation, and virulence of *Escherichia coli* isolates from commercial meat and vegetable products. Foodborne Pathog Dis.

[77] Kumar CG, Anand SK (1998). Significance of microbial biofilms in food industry: a review. Int J Food Microbiol.

[78] Bixler GD, Bhushan B (2012). Biofouling: lessons from nature. Philos Trans A Math Phys Eng Sci.

[79] Awad TS, Asker D, Hatton BD (2018). Food-Safe Modification of Stainless Steel Food-Processing Surfaces to Reduce Bacterial Biofilms. ACS Appl Mater Interfaces.

[80] Kim HW, Oh HS, Kim SR, Lee KB, Yeon KM, Lee CH, Kim S, Lee JK (2013). Microbial population dynamics and proteomics in membrane bioreactors with enzymatic quorum quenching. Appl Microbiol Biotechnol.

[81] Xu C, Lin X, Ren H, Zhang Y, Wang S, Peng X (2006). Analysis of outer membrane proteome of *Escherichia coli* related to resistance to ampicillin and tetracycline. Proteomics.

[82] Altegoer F, Schuhmacher J, Pausch P, Bange G (2014). From molecular evolution to biobricks and synthetic modules: a lesson by the bacterial flagellum. Biotechnol Genet Eng Rev.

[83] Minamino T, Imada K, Namba K (2008). Mechanisms of type III protein export for bacterial flagellar assembly. Mol Biosyst.

[84] Macnab RM (2003). How bacteria assemble flagella. Annu Rev Microbiol.

[85] Lee PC, Rietsch A (2015). Fueling type III secretion. Trends Microbiol.

[86] Manson MD, Tedesco P, Berg HC, Harold FM, Van der Drift C (1977). A protonmotive force drives bacterial flagella. Proc Natl Acad Sci U S A.

[87] Gabel CV, Berg HC (2003). The speed of the flagellar rotary motor of *Escherichia coli* varies linearly with protonmotive force. Proc Natl Acad Sci USA.

[88] Minamino T, Namba K (2008). Distinct roles of the FliI ATPase and proton motive force in bacterial flagellar protein export. Nature.

[89] Bartlett DH, Frantz BB, Matsumura P (1988). Flagellar transcriptional activators FlbB and FlaI: gene sequences and 5' consensus sequences of operons under FlbB and FlaI control. J Bacteriol.

[90] Liu X, Matsumura P (1995). An alternative sigma factor controls transcription of flagellar class-III operons in *Escherichia coli*: gene sequence, overproduction, purification and characterization. Gene.

[91] Macnab RM (1992). Genetics and biogenesis of bacterial flagella. Annu Rev Genet.

[92] Zhao Z, Zhao Y, Zhuang XY, Lo WC, Baker MAB, Lo CJ, Bai F (1885). Frequent pauses in *Escherichia coli* flagella elongation revealed by single cell real-time fluorescence imaging. Nat Commun.

[93] Paul K, Erhardt M, Hirano T, Blair DF, Hughes KT (2008). Energy source of flagellar type III secretion. Nature.

[94] Korea CG, Badouraly R, Prevost MC, Ghigo JM, Beloin C (2010). *Escherichia coli* K-12 possesses multiple cryptic but functional chaperone-usher fimbriae with distinct surface specificities. Environ Microbiol.

[95] Larsonneur F, Martin FA, Mallet A, Martinez-Gil M, Semetey V, Ghigo JM, Beloin C (2016). Functional analysis of *Escherichia coli* Yad fimbriae reveals their potential role in environmental persistence. Environ Microbiol.

[96] Remaut H, Tang C, Henderson NS, Pinkner JS, Wang T, Hultgren SJ, Thanassi DG, Waksman G, Li H (2008). Fiber formation across the bacterial outer membrane by the chaperone/usher pathway. Cell.

[97] Valenski ML, Harris SL, Spears PA, Horton JR, Orndorff PE (2003). The Product of the *fimI* gene is necessary for *Escherichia coli* type 1 pilus biosynthesis. J Bacteriol.

[98] Klemm P, Schembri M: Type 1 Fimbriae, Curli, and Antigen 43: Adhesion, Colonization, and Biofilm Formation. *EcoSal Plus* 2004, 1. 10.1128/ecosalplus.8.3.2.6 26443347

[99] Schwan WR (2011). Regulation of fim genes in uropathogenic *Escherichia coli*. World J Clin Infect Dis.

[100] Inoue T, Shingaki R, Hirose S, Waki K, Mori H, Fukui K (2007). Genome-wide screening of genes required for swarming motility in *Escherichia coli* K-12. J Bacteriol.

[101] Wu XR, Sun TT, Medina JJ (1996). In *vitro* binding of type 1-fimbriated *Escherichia coli* to uroplakins Ia and Ib: relation to urinary tract infections. Proc Natl Acad Sci U S A.

[102] Gumbart J, Wiener MC, Tajkhorshid E (2007). Mechanics of force propagation in TonB-dependent outer membrane transport. Biophys J.

[103] Van Houdt R, Michiels CW (2005). Role of bacterial cell surface structures in *Escherichia coli* biofilm formation. Res Microbiol.

[104] Guttenplan SB, Kearns DB (2013). Regulation of flagellar motility during biofilm formation. FEMS Microbiol Rev.

[105] Wood TK, Gonzalez Barrios AF, Herzberg M, Lee J (2006). Motility influences biofilm architecture in *Escherichia coli*. Appl Microbiol Biotechnol.

[106] Chao Y, Zhang T (2011). Probing roles of lipopolysaccharide, type 1 fimbria, and colanic acid in the attachment of *Escherichia coli* strains on inert surfaces. Langmuir.

[107] Macnab RM (1977). Bacterial flagella rotating in bundles: a study in helical geometry. Proc Natl Acad Sci U S A.

[108] Goryshin IY, Naumann TA, Apodaca J, Reznikoff WS (2003). Chromosomal deletion formation system based on *Tn5* double transposition: use for making minimal genomes and essential gene analysis. Genome Res.

[109] Yu BJ, Sung BH, Koob MD, Lee CH, Lee JH, Lee WS, Kim MS, Kim SC (2002). Minimization of the *Escherichia coli* genome using a Tn5-targeted Cre/loxP excision system. Nat Biotechnol.

[110] Kolisnychenko V, Plunkett G, Herring CD, Feher T, Posfai J, Blattner FR, Posfai G (2002). Engineering a reduced *Escherichia coli* genome. Genome Res.

[111] Lee JH, Sung BH, Kim MS, Blattner FR, Yoon BH, Kim JH, Kim SC (2009). Metabolic engineering of a reduced-genome strain of *Escherichia coli* for L-threonine production. Microb Cell Fact.

[112] Hashimoto M, Ichimura T, Mizoguchi H, Tanaka K, Fujimitsu K, Keyamura K, Ote T, Yamakawa T, Yamazaki Y, Mori H (2005). Cell size and nucleoid organization of engineered *Escherichia coli* cells with a reduced genome. Mol Microbiol.

[113] Mizoguchi H, Sawano Y, Kato J, Mori H (2008). Superpositioning of deletions promotes growth of *Escherichia coli* with a reduced genome. DNA Res.

[114] Hirokawa Y, Kawano H, Tanaka-Masuda K, Nakamura N, Nakagawa A, Ito M, Mori H, Oshima T, Ogasawara N (2013). Genetic manipulations restored the growth fitness of reduced-genome *Escherichia coli*. J Biosci Bioeng.

[115] Lieder S, Nikel PI, de Lorenzo V, Takors R (2015). Genome reduction boosts heterologous gene expression in *Pseudomonas putida*. Microb Cell Fact.

[116] Wang J, Ma W, Wang Y, Lin L, Wang T, Wang Y, Li Y, Wang X (2018). Deletion of 76 genes relevant to flagella and pili formation to facilitate polyhydroxyalkanoate production in *Pseudomonas putida*. Appl Microbiol Biotechnol.

[117] Wang Z, Wang J, Ren G, Li Y, Wang X (2015). Influence of core oligosaccharide of lipopolysaccharide to outer membrane behavior of *Escherichia coli*. Mar Drugs.

[118] Wang Z, Wang J, Ren G, Li Y, Wang X (2016). Deletion of the genes *waaC*, *waaF*, or *waaG* in *Escherichia coli* W3110 disables the flagella biosynthesis. J Basic Microbiol.

